# Exposure to war and conflict: The individual and inherited epigenetic effects on health, with a focus on post-traumatic stress disorder

**DOI:** 10.3389/fepid.2023.1066158

**Published:** 2023-02-16

**Authors:** Zara Raza, Syeda F. Hussain, Victoria S. Foster, Joseph Wall, Peter J. Coffey, John F. Martin, Renata S. M. Gomes

**Affiliations:** ^1^ Research & Innovation, Blind Veterans UK, London, United Kingdom; ^2^ BRAVO VICTOR, Research & Innovation, London, United Kingdom; ^3^ Hull York Medical School, University of York, York, United Kingdom; ^4^ St George’s Hospital Medical School, London, United Kingdom; ^5^ Haxby Group Hull, General Practice Surgery, Hull, United Kingdom; ^6^ Development, Ageing and Disease, UCL Institute of Ophthalmology, University College London, London, United Kingdom; ^7^ Centre for Cardiovascular Biology and Medicine, University College London, London, United Kingdom; ^8^ Northern Hub for Veterans and Military Families Research, Department of Nursing, Midwifery and Health, Faculty of Health and Life Sciences, Northumbria University, Newcastle upon Tyne, United Kingdom

**Keywords:** epigenetics, war, conflict, stress, PTSD, inherited epigenetics, trauma, healthy soldier effect

## Abstract

War and conflict are global phenomena, identified as stress-inducing triggers for epigenetic modifications. In this state-of-the-science narrative review based on systematic principles, we summarise existing data to explore the outcomes of these exposures especially in veterans and show that they may result in an increased likelihood of developing gastrointestinal, auditory, metabolic and circadian issues, as well as post-traumatic stress disorder (PTSD). We also note that, despite a potential “healthy soldier effect”, both veterans and civilians with PTSD exhibit the altered DNA methylation status in hypothalamic–pituitary–adrenal (HPA) axis regulatory genes such as *NR3C1*. Genes associated with sleep (*PAX8*; *LHX1*) are seen to be differentially methylated in veterans. A limited number of studies also revealed hereditary effects of war exposure across groups: decreased cortisol levels and a heightened (sex-linked) mortality risk in offspring. Future large-scale studies further identifying the heritable risks of war, as well as any potential differences between military and civilian populations, would be valuable to inform future healthcare directives.

## Background

The epigenome, a collaborative effort of DNA and its modifications, can be readily modified in response to internal and external changes (1–9). Epigenetics may impact the individual, for example, work-related stress and depression result in altered DNA methylation of the glucocorticoid receptor (GR) gene (*NR3C1*; nuclear receptor subfamily 3 group C member 1), an area well reviewed by Bakusic et al. (10). Epigenetic modifications may be inherited by progeny, and even in the offspring of progeny. Such inheritance is termed “intergenerational”, except in instances where the generation could never have been exposed to stress stimuli (e.g., the grandparent was not pregnant at the time), in which case it is “transgenerational” (11). Non-genetic influence includes the impact of experiences from previous generations, passed down through shared stories or the spoken word, an effect referred to as “intergenerational trauma,” which may contribute to PTSD (12–14).

**Figure 1:**
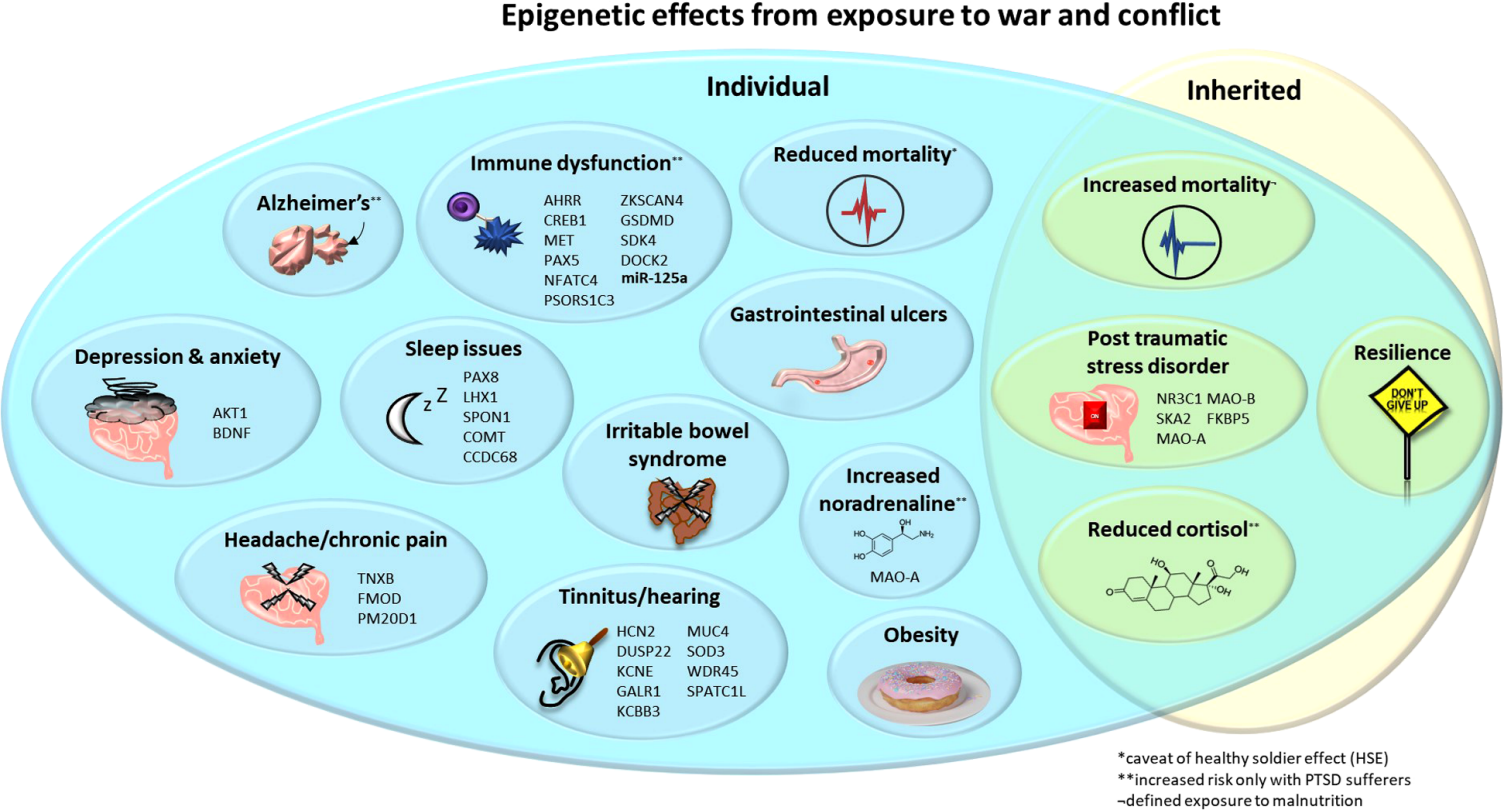
Epigenetic effects from exposure to war and conflict. Upon the individual: decreased cortisol, mortality* and sleep quality/quantity; increased mortality, PTSD, noradrenaline**, tinnitus, immune dysfunction, Alzheimer's disease**, obesity, anxiety, depression, gastrointestinal ulcers and IBS in children exposed to war. Inherited: decreased cortisol and mortality*, and increased risk of PTSD but also resilience. Noted are genes (and the microRNA miR-125a where expression is altered) that are associated with these pathologies and may occur differentially methylated and/or expressed. *Decreased mortality, potentially as a confounding “healthy solider effect”. **Increased risk only in individuals with PTSD. IBS, irritable bowel syndrome; PTSD, post-traumatic stress disorder.

Our review discusses epigenetic impacts and phenotypic outcomes on individuals and families affected by conflict and war, summarised in Figure 1. Exploring whether military populations differ epigenetically in response to conflict when compared to civilians is important. First, as the rigorous military selection process generates a population of highly fit and healthy individuals compared to general society. Second, both military and civilian populations may witness and experience the long-term physical, psychological and economic impacts of war, but it is, in most instances, only military personnel who are expected to actively participate in planned, targeted assault. Therefore, these differential impacts may result in different health outcomes where a one-size-fits-all treatment approach will be ineffective. Current research demonstrates the long-reaching impacts of stressors, such as famine on metabolic, cardiovascular and cerebral health (15). The current global issues, including the war in Ukraine, civil unrest in Iran, upheaval in Afghanistan, conflict in Ethiopia, South Sudan, and Syria (and undoubtably numerous more yet less widely publicised unrest), result in a drive to take in refugees. One review has noted primary healthcare as the prevalent support type used by refugees (98.7%) and found to be the most useful (27.5%) (16). This highlights the necessity to understand epigenetic impacts that may further contribute to, or drive, psychological and/or physiological complications to improve support for vulnerable populations.

## Post-Traumatic Stress Disorder

Hypothalamic–pituitary–adrenal (HPA) axis dysfunction is one of the aetiological factors linked with PTSD (17, 18). The GR, responding to cortisol, is a key player in this axis (19). Increases in cortisol are often associated with higher levels of stress. Paradoxically, patients with chronic PTSD may exhibit lower cortisol, and lowered urinary cortisol after a traumatic event may be predictive of PTSD development (18, 20). It is important to note that cortisol levels are not pathologically low, and still follow a circadian rhythm, but are significantly lower compared to individuals without PTSD (18, 21). In PTSD, a spiking of cortisol levels may also be seen in acute therapy stages, and the administration of a low dose of cortisol may hold promise for alleviation (18, 22). Indeed, administration of dexamethasone (to suppress cortisol) in veterans resulted in a positive correlation between suppression and the severity of PTSD symptoms (18, 23). Yehuda et al. summarise from their research that reduced cortisol may occur due to enhanced negative feedback sensitivity of the HPA axis, i.e., cortisol suppression (18). As an increased number of GRs are seen in individuals with PTSD, the physiological response may still be high despite these lowered levels of cortisol (24).

Altered methylation may result in reduced (or increased) gene expression and, in genes related to the HPA axis, influence the suppression of the sympathetic nervous system. Individuals who have experienced emotional and/or sexual trauma have been reported to show both increased methylation at the promoter region of the *NR3C1* (the GR gene) and reduced messenger RNA (mRNA) levels of the receptor (25, 26). Conversely, another study reported that individuals with lifetime PTSD showed reduced methylation of *NR3C1* at promoter regions and an inverse correlation with overall mRNA expression, which, in turn, inversely correlated with cortisol levels (27). These data reveal the complexity of the HPA axis effects from epigenetic changes; given there are different promoter regions of the gene, this may play a role in differential results. FK506 binding protein 5 (FKBP5) is a negative regulator of GR via the signalling and translocation of glucocorticoids to modulate sensitivity (28) and is also associated with both genetic and environmental modifications (29, 30). Decreased methylation of FKBP5 (predicating increased expression) was found in individuals exposed to early trauma and associated with the GR-induced transcription of FKBP5, resulting in chronic dysregulation of stress hormones (30). Epigenetic marks, i.e., methylation of FKBP5 and/or NR3C1, have been proposed as a possible predictor for the severity of PTSD as well as to define treatment outcome (31–33).

Other genes have also been linked to PTSD symptoms (26). A sex-linked effect for higher levels of circulating peptide [pituitary adenylate cyclase-activating polypeptide (PACAP)], which binds to the pituitary adenylate cyclase-activating polypeptide type I receptor (ADCYAP1R1; PAC-1), was seen for the female symptoms/diagnosis of PTSD (34). Increased methylation of a CpG island of PAC-1 was predictive of PTSD, in a non-sex-specific manner (34) and found in male veterans with PTSD (35). This suggests functionally relevant epigenetic effects may occur as a result of PTSD exposure. Multiple microRNAs (miRNAs) have been shown to play a significant role in the regulation of fear, with differential expression observed in individuals with PTSD (36).

Understanding the role of epigenetics in the pathogenesis of PTSD has allowed for research to be conducted into the therapeutic uses of these epigenetic modifications. A common focus of PTSD treatment is the extinction of memories that cause fear (37). Studies on mice have revealed histone acetylation, methylation, and DNA methylation all contribute to memory extinction (38). Valproic acid (VPA), a histone deacetylate (HDAC) inhibitor, enhances extinction memory for fear associated with audio (39). Murphy and Singewald suggest increasing the occurrence of acetylation promotes de novo transcription, translation and gene expression to allow for the consolidation of long-term extinction memory (36). The use of VPA, an established mood stabiliser for bipolar disorders, has been suggested for the treatment of PTSD alongside psychotherapy (40, 41). Potential treatments, underpinned by epigenetic changes, indicate the importance of understanding these underlying modifications to better aid patients in future.

To summarise, a number of players (e.g., FKBP5, NR3C1, PACAP, PAC-1, and HDAC) can be linked to the pathophysiology of PTSD, likely due to influences on the HPA axis. Next, we will discuss how the trauma of war may evoke these symptoms.

## War and military personnel

Trauma, the exposure to a distressing or disturbing experience, can have long-term negative impacts on individuals that may lead to PTSD (a common diagnosis made in the aftermath of war and conflict) (17, 42). As PTSD-related epigenetic modifications may impact the longevity of individuals, we first evaluate the normal longevity seen in military personnel.

Hartal et al. compared the life expectancy of retired military personnel to the general population and found the mean age at death was greater in comparison to their sex- and birth-matched equivalents in the civilian population. The average life expectancy was found to be exceeded by 67.9% of retired service members (43). The authors attributed many of these results to the “healthy soldier effect” (HSE), which persists even 30 years after service (44), a variation of the “healthy worker effect” (45). This is a tendency for people who work in physically demanding environments—particularly the military—to exhibit lower mortality and morbidity rates relative to the general population, due to the exclusion of those with poor health from participating (46). Soldiers in the US who are wounded in battle receive a Purple Heart (PH) decoration. In veterans aged 65 years and over, PH recipients with or without PTSD showed halved rates of mortality in comparison to those without the award (47). This could be attributed to a “survivor effect,” where the less healthy in this cohort of injured veterans did not survive to this timepoint. Alternatively, veterans who have been injured during combat may receive priority medical care. The epigenetic impact of stressors that military personnel experience may be influenced by these factors.

Verhoeven et al. found the epigenetic age of veterans with PTSD, defined by the methylation of leukocyte DNA, was significantly lower than those without (48). This was partly explained through use of antidepressants, which was associated with reducing telomerase activity and, in turn, genetic aging. However, veterans with PTSD, versus those who have been exposed to trauma but did not have PTSD, have been shown to exhibit the following: (1) increased plasma lipids (49); (2) an exacerbated startle response upon exposure to yohimbine, a noradrenaline inhibitor antagonist (50); and (3) a heightened experience of odour intensity with decreased heart rate in response to burning rubber (a trauma cue) (51).

Therefore, despite a proposed increase in longevity among veterans, the physiological impacts of PTSD persist. We next consider how the epigenetic factors specifically may occur after exposure to conflict.

## Epigenetic impacts of war

Exposure to war results in a plethora of biological impacts, described below and summarised in Figure 1*.* The physiological effects of war trauma seen in veterans with PTSD include co-morbid chronic pain (which may reduce alongside PTSD treatment) (52, 53). These findings were applicable to both male and female veterans (53), although the lack of data pertaining to female veterans often makes sex-specific effects difficult to explore. Other pathologies incurred from war include a higher incidence of peptic ulcers in veterans and increases in dyspepsia and heartburn (2). Australian combat prisoners of war (POWs) in World War II (WWII) Japanese camps exhibited an increased rate of gastrointestinal ulcers (1). Children exposed to war exhibit an increased likelihood of irritable bowel syndrome (IBS), a condition also more common in veteran populations (4). An increased risk of obesity was observed in male veterans but, when adjusted for, were no more likely to exhibit hypertension or diabetes (7).

One group found no change in POW medical mortality (54) but, later, found a clear increase at 5–14 years after WWII, which reduced after this point. This indicates an individual's age at the time of war ending may influence mortality (55). Costa et al. also highlighted an age-related effect; after 30, the age at which a POW became imprisoned resulted in a range of differing effects during ageing. Survivors with the worst conditions showed reduced mortality versus non-POWs (or POWs when conditions were not so poor) 35 years post-imprisonment (56). An established increased risk of mortality through ischemic heart disease was found to be even more prevalent after 75 years of age (57, 58). POWs aged younger than 30 years showed an increased risk of cardio- and cerebrovascular disease as well as morbidity from the former (56). As an increased risk of cardiovascular disease is also seen in those prenatally exposed to famine, the malnutrition experienced by POWs may therefore convolute specific impacts of war (e.g., combat exposure) (59, 60). During WWII, the genocide of six million European Jews and other ethnic and societal minorities occurred. Studies on individuals exposed to the Shoah provide information on those experiencing the effects of war through torture, and genocide, but also severe famine. Despite these impacts, small-scale Holocaust studies did not reveal an increased risk of mortality at two timepoints [20–41 (61) and 40–50 (62) years after WWII].

As with other civilian populations, veterans (as well as those exposed to the Holocaust 63), despite often showing increased resilience, are at risk of mental health conditions such as depression and anxiety, which negatively impact on quality of life (64–69). A reduced expression of brain-derived neurotrophic factor (BDNF) is exhibited in depression (70) and has been found to be dysregulated in PTSD/traumatic brain injury (TBI) in veterans (71). In addition, one study revealed the increased methylation of the BDNF promoter region is associated with a PTSD diagnosis in Vietnam War veterans (72). Furthermore, older veterans with PTSD exhibit a twofold increase in the risk for dementia (73, 74). Veterans showed increased levels of depression 40 years on compared to non-POWs; however, in this study, there were no differences in alcohol issues or anxiety (54).

As mentioned above regarding civilian groups with PTSD, lowered cortisol has also been shown in combat veterans and Holocaust survivors with PTSD (75–77). The hormone and neurotransmitter noradrenaline, the mediator of the HPA-stress system, was present at the baseline in higher levels in the cerebrospinal fluid (CSF) from male combat veterans with PTSD, an effect not replicated in blood plasma (78). Blood plasma again was reported by Yahyavi et al. to show normal levels of adrenaline and noradrenaline in the plasma of veterans with PTSD (and their offspring) but decreased cortisol in those with PTSD (79). A study using CSF samples from 52 veterans with trauma revealed no significant difference in noradrenaline levels between those with or without PTSD/trauma when taking the medication prazosin (which treats high blood pressure and heart failure). However, in those not taking the drug, a significantly positive correlation between behavioural symptoms (e.g., depression and insomnia) and higher noradrenaline levels was revealed (80). The authors suggest trauma exposure may influence responsiveness to noradrenaline, and these data reveal other medication may influence hormone levels.

Above, we have discussed the physiological ramifications of war and conflict exposure, such as gastrointestinal upset (included in Figure 1). We will further expand upon this in the next section while specifically discussing epigenetic alterations (summarised in Table 1), predominantly pertaining to gene methylation.

**Table 1:** Exposure to war and subsequent effects on epigenetics to individuals measured through gene methylation.

Study	Gene implicated and methylation/expression status			
Hammamieh et al. (81); veterans with PTSD (*n* = 99) and without (*n* = 101). Age range 20–60 years. Cohort of men only. Individuals with TBI, neurologic disorder and psychiatric history excluded. Blood samples showing DMGs.	**AKT1**↑: network linked to anxiety and depression.			
	**BDNF**↑: expression high in serum and low in plasma.^a^			
	**CNR1**↑: SNP variants of this gene linked to PTSD.			
	**CREB1**↑: changes to expression seen in monocytes of individuals with PTSD.			
	**EFS**↑: —			
	**ETS-2**↑: genes associated with growth and development.			
	**HES4**↑: —			
	**LHX1**↑: circadian rhythms (82).			
	**MET**↑: immune system signalling (83).			
	**NR2E1**↑: loss of gene makes mice very aggressive (erroneously stated under human studies in original table) (81).			
	**PAX5**↑: immune system (84).			
	**PDGFB**↑: platelets.			
	**PSD**↑: —			
	**TRERF1**↑: —			
	**PTTG1IP**↓: —			
	**NFATC4**↓: T-cells (85).			
	**GATA3**↓: —			
	**ELK1**↓: —			
	**DMRTA2**↓: —			
Wang et al. (6); Participants from US Army explosive entry training sites (*n* = 34); male cohort; mean age 30.79 years; 60% reported mild TBI. Blood samples before and after training with exposure to blasts. Only results seen in cumulative lifetime exposure, not pre- and post-training.	Cumulative exposure to blasts (e.g., high blast exposed groups). Significantly DMRs and reported gene expression	**PAX8**↑: downregulated expression. Sleep.		
		**SLFN3**↓: no change in expression.		
		**LOC643387**↑: downregulated expression.		
		**DSCR3**↑: no change in expression.		
		**SFAM53A**↓: increased expression.		
		**NRP2**↑: downregulated expression.		
		**NPHP1**↑: downregulated expression.		
	Notable symptom-associated DNA methylation analyses with reported gene expression; did not show statistically significant genome-wide DMRs that tracked with reported symptoms of headache post-blast exposure, after multiple testing correction.	**HCN2**↑: expression not reported; prior implication in auditory function.		
		**DUSP22**↓: no change in expression; prior implication in auditory function.		
		**KCNE**↑: downregulated expression. Associated with hearing loss.		
		**CYP2E1**↑: downregulated expression. Rodent association with hearing loss.		
	**KCTD12**: no reported change to methylation. Downregulated expression; association with tinnitus.			
Wang et al. (5); military trainees; varying lifetime histories (*n* = 59). Only results seen in cumulative lifetime exposure. DNA methylation in blood samples.	Six significantly DMRs identified using the combined *p*-value tool in high relative to low-blast exposed groups	**PSORS1C3**↑: Autoimmune disorders, under glucocorticoid control (86).		
		**GSDMD**↑: IL-1 secretion (87).		
		**NTSR1**↑: dysregulated sleep and circadian rhythms.		
		**SPON1**↓: dysregulated sleep and circadian rhythms.		
		**ZKSCAN4**↓: Associated with autoimmune disorders.		
		**ACSM6**↓: —		
	Symptoms seen in this study associated with DNA methylation of known genes shown in high cumulative exposed groups noted in the paper.	**COMT**↑: sleep disturbance.		
		**CCDC68**↑: sleep disturbance.		
		**TNXB**↑: headache (associated with chronic pain, temporomandibular joint dysfunction).		
		**FMOD**↓: headache (gene associated with chronic pain, temporomandibular joint dysfunction).		
		**GALR1**↑: tinnitus (auditory dysfunction)		
		**KCNN3**↓: tinnitus (auditory function).		
		**MUC4**↓: tinnitus (hearing loss).		
		**SOD3**↓: tinnitus (hearing impairment).		
		**WDR45**↓: tinnitus (auditory dysfunction).		
Rutten et al. (88); Male Dutch military servicemen post deployment; Group 1: High PTSD symptoms and high combat trauma exposure, *n* = 32; Group 2: low PTSD symptoms and high combat trauma exposure, *n* = 29 and Group 3: low PTSD symptoms and low combat trauma exposure. Mean age 27.5 years. Study and replicated in male US marines (*n* = 98). Blood samples.	DMPs direction of effect for all identified DMPs was negative (e.g., increased PTSD symptoms associated with decreased DNA methylation over time). We include associated links of these genes.		**DUSP22**↓: replicated but opposite directionality	
			**PAX8**↓: sleep (6).	
			**NINJ2**↓: nerve injury (89).	
			**HOOK2**↓: —	
			**COL1A2**↓: collagen.	
			**HIST1H2APS2**↓: histones; replicated in second data set.	
			**SDK1**↓: neurological conditions (90) replicated but opposite directionality.	
			**MYT1l**↓: neurological conditions (91).	
	The strength of the observed associations between traumatic stress and PTSD symptoms was mediated by DNA methylation changes of seven DMRs. Indication of increased PTSD symptom scores over time associated with decreased DNA methylation levels at the DMR. We include associated links of these genes.		**RNF39 ↓** associated with an increase in PTSD symptoms over time. Replicated in second data set.	
			**HOOK2**↓: —	
			**PAX8**↓: sleep.	
			**SPATC1l**↓: hearing loss.	
			**PM20D1**↓: Alzheimer's disease (92).	
			**SMAD5**↓: haematopoiesis (93).	
			**GPR12**↓: neuronal development (94).	
			**ZFP57**↓: role in DNA methylation (95). Replicated in second data set.	
Sadeh et al. (96); white non-Hispanic service members (*n* = 200); consecutively enrolled in the Translational Research Center for Traumatic Brain Injury and Stress Disorders. Members excluded from neuroimaging but not blood methylation measurements (*n* = 55) due to moderate/severe TBI history.	**SKA2↑**: correlation of methylation (adjusted for phenotype) with increased PTSD severity and associated with reduced cortical thickness in prefrontal cortex. SKA2 is a potential indicator for suicide risk.			
Smith et al. (97); Ten cohorts, military and civilian, contribute blood-derived DNA methylation data from PTSD cases and trauma-exposed controls (*n* = 1,896). Mixed gender, black, white and Hispanic military (*n* = 1351) and civilian (*n* = 545) populations.	**AHRR**↓: In individuals with PTSD vs. trauma-exposed controls. Decreased methylation also associated lower kynurenine levels in individuals with PTSD, evident in non-smokers, suggesting an underpinning factor for immune dysregulation.			
Ziegler et al. (98); patients with current PTSD (*n* = 195; 140 men); remitted PTSD (*n* = 136) and healthy controls (*n* = 321). Blood samples for methylation analysis. Mean age approximately 49 years.	**MAO-A**↑: at three CpG sites only in men vs. remitted and healthy controls. Potential tool for assessment of PTSD severity post-war. Gene involved in catabolising noradrenaline.			
Zhou et al. (99) Combat veterans with PTSD (*n* = 30; men = 27) and control group without. Age range 29–67 years. Blood samples.	Many microRNAs upregulated in PTSD. Also in PTSD groups, downregulation of miR-125a (as well as miR-181c); inhibitory effect of miR-125a on IFN-γ release in vitro*.* Interferon gamma (IFN-γ) and IL-17 were also found in the plasma. Significant increase of peripheral blood mononuclear cell numbers in PTSD patients vs. controls, which also correlated with anxiety. Supports immune dysregulation in PTSD.			
Yehuda et al. (31); veterans with PTSD received PE psychotherapy, responders (*n* = 8, age approximately 41 years) and non-responders (*n* = 8, age approximately 58 years). Significant difference in ages. Two women in cohort.	**FKBP5**↓: decreased in association with recovery from PTSD (e.g., successful treatment), and higher gene expression observed in a subset of responders during follow-up. Higher methylation associated with lower plasma and urinary cortisol before treatment.			
	**NR3C1** (**GR**; exon 1F promoter) ↑: increased methylation before treatment predicted positive outcome (e.g., responders) and positively associated with post-treatment urinary cortisol. No changes to methylation associated with post-treatment or follow-up.			
Yehuda et al. (100); male combat veterans with PTSD (*n* = 61) and without PTSD (*n* = 61). Age approximately 34 years. Blood samples for plasma for cell counts (PBMCs) and DNA methylation. Urine samples for cortisol.	**NR3C1** (GR; exon 1F promoter) ↓: in PBMCs from combat veterans with PTSD compared with combat-exposed veterans who did not develop PTSD. Methylation inversely correlated with clinical markers and symptoms associated with PTSD. This was also associated with functional measures such as suppressed lysozyme (in vitro measure of GR sensitivity) and decreased urinary cortisol.			
Mehta et al. (35); Australian male veterans from the Vietnam War with PTSD (*n* = 48) and without (*n* = 48); age range 62–88 years. Replicated in a population of primarily African American men from the Grady Trauma Project (*n* = 115). Cross-sectional association study. Blood samples.	Candidate genes significant, after multiple testing correction associated with symptom severity. Of the genes, 43% identified with initial CpG significance (including FKBP5, NR3C2, RORA) were initially significant, but not after correction. No further information from authors on which genes are hyper/hypomethylated.			**ADCYAP1R1 (PAC-1)**
				**ANK3**
				**BDNF**
				**CNR1**
				**COMT**
				**CRHR1**
				**CRHR2**
				**DRD2**
				**GR/NR3C1**
				**MAOA**
				**MAOB**
				**NOS1AP**
				**NPY**
				**SLC6A3**
				**STMN1**
				**TPH1**
				**WWC1**
	Genome-wide DNA methylation in PTSD among combat veterans, associated with increased symptom severity.			**Intergenic CPG** (43 kb from LRRC3B) ↓:
				**BRSK1**↑:
				**NGF**↑:
				**LCN8**↑:
				**DOCK2**↑: role in immune system and neurodegeneration.
Kim et al. (72); male combat veterans with PTSD (*n* = 126) and without (*n* = 122). Mean age approximately 63 years. Peripheral blood to detect DNA methylation. Participants with a history of head trauma were excluded.	**BDNF**↑: at four CPG sites, in individuals with PTSD vs. those without PTSD. PTSD diagnosis significantly associated with high BDNF methylation, high combat exposure and issues with alcohol.			
Sarapas et al. (101); survivors of the 9/11 attacks with and without PTSD. Individuals recovered from PTSD are also included. Whole blood gene expression and cortisol levels as well as genome-wide gene expression was analysed. 25 probe sets were differentially expressed in PTSD.	**STAT5B**: reduced expression.			
	**Nuclear factor I/A**: reduced expression.			
	**FKBP5**: reduced expression.			
	**MHC Class II**: reduced expression.			

BDNF, brain-derived neurotrophic factor; DMG, differentially methylated gene; DMP, differentially methylated position; DMR, differentially methylated region; GR, glucocorticoid receptor; PBMC, peripheral blood mononuclear cell; PE, prolonged exposure; PTSD, post-traumatic stress disorder; SNP, single nucleotide polymorphism; TBI, traumatic brain injury.

Citations included for implicated pathologies if not referenced in original study paper. Hypermethylated: ↑, hypomethylated: ↓.

^a^ The authors note the plasma result was not validated in the later study. Human studies unless otherwise stated.

Monoamine oxidase A (MAO-A) is an enzyme critically involved in catalysing adrenaline, noradrenaline, dopamine and serotonin (102). Hypermethylation of the *MAO-A* gene is linked to PTSD (35) and was suggested as a tool to assess the severity of the disorder in male patients with PTSD due to war exposure; a sex-linked effect was seen, although the female sample size was comparatively small (see Table 1) (98). Gene hypermethylation often results in reduced expression, in this case potentially impeding catabolic activity on noradrenaline resulting in increased circulating levels upon stress exposure. Such dysregulation of stress hormones is a hallmark of PTSD.

In combat veteran studies, those with PTSD exhibited NR3C1 hypermethylation when compared to those without PTSD, an effect not seen in those exposed to childhood trauma (31, 35, 100). The impact of the Tutsi genocide in Rwanda was studied in 25 pregnant women and found higher CpG methylation in the nuclear receptor subfamily 3 group C member 2 (*NR3C2*; a mineralocorticoid receptor, also binds glucocorticoids) gene (103). The increased methylation of spindle and kinetochore-associated protein 2 (SKA2) plays a role in GR chaperoning, providing negative feedback to the HPA by removing the receptor from the cytoplasm and to the nucleus (104). Blood methylation of SKA2 and reduced expression, along with aberrant glucocorticoid signalling, was identified as a potential indicator for suicide risk (105, 106). In veterans, a positive correlation of SKA2 methylation in the blood was seen with severity of PTSD symptoms (96).

Sleep difficulties are often reported by veterans (8), including those with PTSD (107), making these epigenetic changes an area of interest. Indeed, an increase in methylation on the circadian clock gene, *LHX1*, has been seen in veterans with PTSD (81). Impacts on sleep-related genes were found in military personnel with chronic blast exposure, including differential methylation of *NTSR1* and *SPON1* (5), and differentially methylated regions (DMRs) for *CCDC68* and *COMT* (also linked to sleep difficulties experienced by the individuals). An earlier study by the same group found, concurrent with impacts on sleep, chronic exposure to blasts resulted in the increased methylation of the paired box gene 8 (*PAX8*) antisense transcript, related to repressed gene expression (6). However, another study found decreased PAX8 methylation in veterans with PTSD (88). PAX8 is associated with thyroid function (108) as well as sleep duration (109). Sleep after exposure to emotional information was shown to consolidate memory up to 4 years later, so initial sleep disruption may be beneficial to reduce PTSD severity (110). However, this is a short-term benefit, given that persistently poor sleep impedes mental health (111). To alleviate PTSD, attempts to restore effective sleep should be undertaken; gene methylation may offer a useful marker of success.

Chronic blast exposure also revealed the differential methylation of auditory function genes (KCNN3, SOD3, MUC3, GALR1 and WDR45B) which were associated with tinnitus, and the DMRs within FMOD and TNXB were linked to headache and pain (5). Symptoms of tinnitus in low versus high blast exposure groups were also linked to DMRs in auditory genes *KCNE1* and *CYP2E1*, showing an inverse correlation with methylation and expression (6). Finally, pre- versus post-blast exposure showed at least a 1.5-fold difference in expression in 67 genes, such as *UFC1* and *YOD1* (ubiquitin related proteins; others have been linked to TBI). The dysregulation of cytokine and chemokines was also exhibited in MCP-1, GCSF, HGF, MCSF, and RANTES after acute exposure to blasts (6). Blast exposure can result in neurological impacts, such as brain swelling and neurotrauma; these epigenetic markers are evidence of both the psychological and physical impacts of war (5, 6).

PTSD has been associated with increased immunological inflammation (112). A number of immunological changes were characterised in a study on combat veterans with PTSD (99). First, there was a significant increase in the numbers of (1) T-helper 1 (Th1; CD4^+^), (2) Th17, (3) cytotoxic T (CD8+), and (4) B cells. It was found that an increase in Th1 percentage was correlated with severity of PTSD score, as well as a reduction in the number of T-regulatory cells. These data suggest a heightened inflammatory response with reduced regulation. Reflecting this, upregulated levels of the proinflammatory cytokines interferon gamma (IFN-γ) and IL-17 were seen in the plasma. Zhou et al. then revealed that these changes were associated with the downregulation of the miRNA, miR-125a (as well as miR-181c), and went on to show the inhibitory effect of miR-125a on IFN-γ release in vitro *(*99). This signalling molecule has been linked to the upregulation of another proinflammatory cytokine, NF-κB, in human cancer cell lines (113). In rats, NF-κB is necessary for memory reconsolidation (e.g., to allow for alterations of negative associations) (114, 115). This suggests that the cytokine cascade in PTSD is dysregulated. Regular amounts of miR-125a would control the inflammatory response; by reducing IFN-γ and increasing NF-κB, a reduction of trauma responses may occur.

Further exploring the immunological impacts, the largest epigenetics study to date was performed on mixed veteran and civilian populations with and without PTSD (see Table 1) (97). The aryl-hydrocarbon receptor repressor (AHRR), a gene that regulates transcription, revealed four CpG sites in which DNA methylation was lower in individuals with PTSD versus the controls. This was found to be associated with the reduced kynurenine levels also seen in individuals with PTSD (97). The authors suggest that the reduced levels of kynurenine (a metabolite that promotes T-regulatory cell differentiation) may explain why increased inflammation is seen in individuals with PTSD. This form of gene methylation appears in both military and civilian populations with PTSD, which suggests that the HSE is not evident in this instance.

Differential expression of 25 genes associated with HPA, immune and/or cerebral function was seen in survivors of the 9/11 attack with PTSD (101). The altered expression of *FKBP5* was revealed as an acute state marker for PTSD as well as major histocompatibility complex (MHC) Class II (vs. lifetime PTSD). In addition, the reduced expression of Stat five B inhibitor (STAT5B; a GR inhibitor 116) and nuclear factor I/A (key for astrocytic function 117) was seen in PTSD. These genes reveal that epigenetic impacts from PTSD impact on both the HPA axis and the immune system.

Rutten et al. compared the longitudinal changes of genome-wide blood DNA methylation profiles of two different subgroups of soldiers and identified the genes associated with stress in a military context (included in Table 1) (88). In the first cohort, consisting of Dutch military personnel, they discovered 17 differentially methylated positions (DMPs) and 12 DMRs in individuals after they had been deployed to a combat zone. The DMPs and DMRs were then used to explore replication in a different cohort of US marines. In this group, a decrease in DNA methylation at the genes *ZFP57*, *RNF39*, and *HIST1H2APS2* was associated with an increase in PTSD symptoms over time. They found that the association between trauma exposure and PTSD symptoms was mediated by DNA methylation; those who did not exhibit PTSD symptoms showed increased methylation, but those who did develop PTSD symptoms exhibited decreased DNA methylation at the DMP and DMRs. Altered methylation on *DUSP22* and *PAX8* genes, earlier identified, revealed decreased methylation in both, with no change in expression to the former but increased expression in the latter (6, 88).

PTSD is often defined solely by symptom severity, despite the heterogenous nature of the symptoms themselves. To challenge this perception, Yang and colleagues defined the epigenetic biotypes of veterans/active-duty personnel and propensity for PTSD (118). They revealed two main subsets with opposing symptoms: G1 (faster recovery from PTSD, lower methylation vs. controls) and G2 (increased risk of PTSD, higher methylation vs. controls), enriched for individuals also exhibiting major depressive disorder (MDD). That study highlights the complex aetiology of PTSD and variation within military subsets. However, the authors did not discuss immunological components or pro-inflammatory processes, which also contribute to PTSD (99, 112, 119). The identification of if, and how, G1 and G2 express different immunological profiles would inform on whether immune function also differs with opposing symptoms.

Therefore, there is a large body of research exploring a plethora of epigenetic changes upon the individual exposed to war (listed with details in Table 1), which we also attempt to summarise here. As noted earlier, the *FKBP5* and *NR3C1* genes are epigenetically modified; in addition, research on war and conflict exposure has revealed epigenetic impacts to players in auditory, sleep and immune systems.

## Intergenerational impacts of war

Paternal trauma experienced by the survivors of Confederate POW camps, during the US Civil War (1861–1865), was found to impact the life expectancy of their children (see Table 2) (120). During the first 2 years of conflict, there was an exchange period during which prisoners were swapped between the warring sides. However, a “no-exchange period” period occurred between July 1863 to 1864, during which increased camp populations resulted in worsened conditions and many resultant deaths of POWs on both sides. It was found that sons born post-war to POWs who experienced the no-exchange period were 9% and 11% more likely to die early in comparison to sons of exchange-period POWs and non-POWs, respectively. Within families, when comparing sons born pre-war to those born post-war from no-exchange POW fathers, the latter were 2.23 times more likely to die early. However, in this comparison, the authors note the caveat of small sample size. No significance was seen on the daughters of POWs. Post-war factors, such as paternal socioeconomic status, did not impact these results. The sex-specific effects seen in this study population are similar to an effect observed in the Överkalix population of Sweden, where grandparents experiencing an abundant harvest resulted in an increased risk of cardiovascular disease and reduced longevity (131, 132). As in the Överkalix studies, the authors propose that the epigenetic response is transmitted via the Y chromosome, which could explain why the effects are seen only in sons.

**Table 2:** Exposure to war and subsequent inherited epigenetics to offspring.

Study details	Outcome
Yehuda et al. (121); Holocaust survivors (*n* = 32), adult offspring (*n* = 22), control parents (*n* = 8), their offspring (*n* = 9).	Correlation of increased methylation of FKBP5 in Holocaust survivors vs. decreased methylation in their offspring.
Yehuda et al. (122); adult offspring of Holocaust survivors (*n* = 211), demographically comparable Jewish controls (*n* = 73); subdivided on parental lifetime PTSD status.	Overall higher prevalence of mood, anxiety disorders, substance abuse and lifetime PTSD seen in Holocaust survivors' offspring. Maternal PTSD made a larger contribution to PTSD risk in offspring.
Yehuda et al. (76); adult offspring of Holocaust survivors (*n* = 35), healthy comparison controls (*n* = 15); PTSD and parental PTSD status; 24-h urinary cortisol levels measured.	Significant association of low cortisol with PTSD in parents and lifetime PTSD in individuals. Lowest cortisol seen in offspring with lifetime and parental PTSD.
Yehuda et al. (123); adult offspring of Holocaust survivors with parental PTSD (*n* = 13), adult offspring of Holocaust survivors without parental PTSD (*n* = 12); controls (*n* = 16); blood cortisol after dexamethasone suppression.	Increased suppression of cortisol via dexamethasone administration predominantly linked to status of parental PTSD.
Yahyavi et al. (79); veterans with PTSD (*n* = 41), their offspring (*n* = 41), veterans without PTSD (*n* = 43), their offspring (*n* = 43); afternoon serum cortisol recorded.	Offspring of veterans with PTSD showed decreased cortisol, only when groups arranged to show PTSD history. No changes to adrenaline or noradrenaline.
Yehuda et al. (124); women pregnant and present in World Trade Center attack (11 September 2001) (*n* = 38) and 1-year-old babies; salivary cortisol samples.	Significantly lower levels of cortisol in mothers with PTSD and their babies; most significant in babies when exposed during third trimester.
Yehuda et al. (125); adult offspring of Holocaust survivors (*n* = 39) with parental and/or lifetime PTSD, healthy comparison controls (*n* = 15); urinary cortisol.	Both parents must be affected with PTSD for offspring association with lower cortisol. Significant negative correlation seen for severity of parental PTSD accounting for offspring urinary cortisol, as well as offspring PTSD and said levels. No effect on age or gender.
Yehuda et al. (126); adult offspring of Holocaust survivors with parental PTSD (*n* = 23), 10 comparison controls with non-exposed parents (*n* = 10); blood cortisol. No participant had PTSD.	Lower mean and amplitude of cortisol in offspring with parental PTSD vs. without, and offspring of non-exposed parents. Associated sex-specific (maternal PTSD) risk factor.
Yehuda et al. (127); prevalence of PTSD and other psychiatric diagnoses in adult offspring of Holocaust survivors (*n* = 100), comparison controls (*n* = 44). Recruited from clinical and non-clinical populations.	Adult offspring of Holocaust survivors show increased prevalence of PTSD and psychiatric diagnoses (depression, anxiety, substance abuse, eating disorders).
Yehuda et al. (128); adult Holocaust survivors (*n* = 22) and their 22 offspring (*n* = 22).	Increased likelihood of development of PTSD in offspring with traumatic events if parents had PTSD.
Perroud (103); 25 women and offspring exposed to Tutsi genocide vs. 25 non-exposed women and children of the same ethnicity.	Higher peripheral blood methylation in the exon 1F promoter of NR3C1 in mothers and offspring (methylation higher in NR3C2 in mothers) and reduced cortisol levels in mother and child.
Costa et al. (120); children born after the US Civil War (1861–1865) to survivors of Confederate POW camps; children (*n* = 2,342) of no-exchange period ex-POWs (*n* = 732), children (*n* = 2,416) of exchange-period ex-POWs (*n* = 715), children (*n* = 15,145) of non-POW veterans (*n* = 4,920). All born after 1866, surviving to age 45 years.	Sex-specific impacts from father to son; no impact seen on daughters. Sons born post-war to POWs who endured prison during the no-exchange period were 11% more likely to die early vs. non-POW sons and 9% more than sons of exchange-period POWs. Within families, sons of no-exchange ex-POW fathers born after the war died at 2.23 times the rate of those born before the war.
Solomon et al. (129); Israeli combat participants in 1982 Lebanon war, offspring of Holocaust survivors (*n* = 44), offspring of non-Holocaust survivor parents (*n* = 52). PTSD inventory scores taken from questionnaires.	Higher rates of PTSD in the Holocaust survivor children cohort, 1–3 years after participation in the Lebanon war. Suggested that recovery from PTSD was also slower in veterans with Holocaust survivor parents.
Mulligan et al. (130); women from the Democratic Republic of Congo who had experienced war and violence in 2010 (*n* = 25). Whole blood and umbilical cord blood samples.	Methylation of NR3C1 in new-born infants correlated with increased severity of war stress. Also decreased birthweight correlated with increased NR3C1 methylation.

POW, prisoner of war; PTSD, post-traumatic stress disorder.

This includes study details and descriptive outcomes including both alterations to genes, as well as physiological effects.

One recurrent effect noted in studies on the offspring of Holocaust survivors with PTSD was the impact of reduced cortisol levels and an increased risk of PTSD, with an association between the two (76, 123, 125–129). These data have been highlighted with the caveat of the family environment, which may influence behaviour and mindset. However, for Holocaust survivors, in keeping with the HPA axis effects, glucocorticoid sensitivity was increased in the offspring with maternal exposure to PTSD, yet decreased with paternal exposure, effects not influenced by parental care (122, 133). The children of Holocaust survivors exhibit either no prevalence towards psychiatric disorders (134), or in another study, higher instances of mood and anxiety disorders (122). A decreased cortisol effect was present in the offspring of veterans, but only those with a parental history of PTSD (79).

Individuals exposed to the World Trade Centre attacks produced both children and grandchildren who exhibit lower levels of salivary cortisol (124). The data are referred to as transgenerational, but, as grandmothers were pregnant at the time of the attacks, this, by definition, is an intergenerational effect.

Epigenetic effects via gene methylation on the offspring of those exposed to conflict have largely been shown in genes already associated with PTSD. The study discussed earlier by Perroud et al. on mothers exposed to the Tutsi genocide also explored impacts to offspring; both groups showed higher levels of PTSD and decreased cortisol in comparison to controls. Differential methylation was seen, with elevated methylation of *NR3C1* (promoter region) seen only in offspring (vs. the increased level of *NR3C2* methylation in mothers) (103). Prenatal exposure to maternal depression/anxiety has been associated with increased cortisol stress response at 3 months of age, and an associated increase of *NR3C1* (predicted NGFI-A binding site) methylation (135). In the eastern Democratic Republic of Congo, mothers and their offspring in utero were exposed to the stressors of war. The offspring exhibited methylation on the *NR3C1* (promoter region) gene, which differed from the mother, and correlated with prenatal exposure to stress (130). Finally, Yehuda et al. revealed, when compared to controls, enhanced methylation of *FKBP5* (intron 7) in Holocaust survivors versus the lower levels of methylation in their offspring, outcomes that were significantly correlated (121). Differential methylation patterns between parent and offspring have been suggested as a form of compensation for the trauma experienced by the parents (136), further supported by a meta-analysis finding children of Holocaust survivors were generally well adapted, with little evidence to suggest secondary traumatisation (137).

To conclude this section, in comparison to the epigenetic effects impacting the individual, such as DNA methylation on a number of genes, data on inter- and transgenerational epigenetic effects of war and conflict are mainly limited to the well-established *NR3C1* and *FKBP5* genes, mortality, cortisol levels and risk of PTSD (summarised in Table 2). This highlights a large gap in the field; we do not know whether the progeny of those exposed to war may also exhibit higher risks of pathologies, such as sleep disruption, and metabolic and/or gastrointestinal effects as seen in their parent/s. Immunological dysfunction is linked to the HPA axis (138) and PTSD (112), so lowered cortisol levels in the progeny of those exposed to war could result in impacts on the immune system. To improve the health and well-being of those exposed to war, and their families, such effects should be defined to allow for appropriate and timely intervention.

## Discussion

The present article shows that stressors associated with war and conflict have epigenetic impacts on health, at individual, inter- and/or transgenerational levels. Individual impacts uninfluenced by PTSD may present as gastrointestinal (1–4), auditory (5, 6), metabolic (7) and circadian (8) effects (see Figure 1). Shared effects between individuals and their progeny include an increased risk of PTSD (and co-occurring lowered cortisol), as well as changes to mortality; in some cases, these effects are sex-linked (120). Persistent epigenetic markers across generations exposed to war are seen in *NR3C1*, *NR3C2*, and *FKBP5* genes, known players in the HPA axis. Future work may wish to consider the inter- and transgenerational inheritance of other genetic factors shown in Figure 1, such as whether AHRR methylation or reduced miR-125a expression in veterans (99) also occurs in the progeny of veterans and trauma-exposed civilians. We do not know what the long-term effects of the numerous conflicts mentioned in our introduction (e.g., the war in Ukraine or in Iran with the aggressive government measures against citizens during civil unrest) will be. However, we propose that there may be an increased risk of the pathologies described above. Thus, when providing effective international aid and support to individuals seeking refuge in the UK, being cognisant of such downstream epigenetic effects could inform mitigation strategies through short- and long-term health and social care. The British Medical Association (BMA) currently suggests a number of guidelines, named “Refugee and Asylum Seeker Health Toolkit,” which include issues refugees may have in general regarding the control of blood sugar and advises screening for communicable diseases. The GOV.UK “Migrant Health Guide” also provides specific guides for nutrition and mental health support, noting impacts of depression and anxiety. This highlights a focus on PTSD, recognising the necessity of addressing the pathology. We would propose additionally considering gastrointestinal dysfunction, impacts to hearing due to explosives and attempts to provide education on sleep.

We note potentially confounding (but unavoidable) factors from the sequalae of war. First, TBI from military trauma is known as a causative factor for PTSD and/or dementia (107). This therefore includes a psychological and physical influence upon PTSD. Another issue is concurrent malnutrition experienced in POW studies; famine results in cardiovascular impacts and the sole study revealing a cardiac effect (ischaemic heart disease) where POWs were reported as experiencing severe malnutrition (57, 58). Noxious auditory stimuli (e.g., blast exposure) may vary depending on the war environment, or job role within the military, and significant epigenetic effects occur with lifetime blast exposure. Age may also impact the prevalence of morbidity (55, 56). We mention the HSE influencing increased longevity in military personnel, but in more recent populations this effect appears eroded (139). As noted in Tables 1 and 2, most war-related studies have been conducted on Caucasian male veterans or African American individuals who have experienced trauma. More diverse studies, such as that of Smith and colleagues, are required to truly understand the possible contribution of factors such as race, sex, and cultural experiences (97). Despite the unique nature of military populations, many PTSD-linked alterations appear similar across veteran PTSD and PTSD linked to non-military adult trauma, e.g., AHRR methylation as a predicator for PTSD in both military and civilian populations (97). Thus far, when considering exposure to war, there is limited evidence to suggest, without PTSD or exposure to famine, that veterans specifically transmit substantive epigenetic impacts.
